# The Future of Voluntary Hospitals

**Published:** 1923-02

**Authors:** 


### THE FUTURE OF VOLUNTARY HOSPITALS.

### WHAT THE NEW FINANCE MAY DO.

In our December number Mr. E. W. Morris, the House Governor of the London Hospital, gave a general account of the Hospital Saving Association Scheme. We now supplement this outline by some extracts from a series of valuable "Notes on Contributory Schemes" drawn up by Mr. Morris and Mr. N. Langton, the Appeal Secretary of the Hospital.

### The Merging of Giver and Receiver.

Hitherto it has been acknowledged that a voluntary hospital is a charity, and that it deals with two classes of the public—those who support the charity and those who benefit by the charity—the givers and the receivers. And these two classes have been kept apart, at any rate officially. It has always been recognised that the giver gives, not for his own good, but for someone else's, and that the receiver receives because someone else has paid the cost. The Notes continue :—

Many things have happened in more recent years which have tended to obliterate this sharp line of distinction between donor and recipient, and we seem to be approaching the time when the giver and the receiver may be one and the same person. The principal cause for this coming change is the increasing cost of hospital administration. The oldest minutes spoke of "generous donors" and of "miserable objects." These two classes are merging, and the generous donor of one year may be the miserable object of next. The natural result is that the giver, the small giver, at any rate, while quite willing to give for the general good, feels that he himself should be able to benefit in the general good should occasion arise. The tendency receives a "push" from another cause. The social standing of the hospital patient has improved since the hospitals were established. The class for which they originally existed now go to the infirmary (Poor Law), and consequently the hospital bed is now often filled by a patient who has subscribed to the hospital for years, and now, as a patient, would prefer to contribute towards part of his cost while an inmate.

### The Hospital as Sick Club.

The London hospitals are turning to a contributory scheme partly because the finding of a guinea a week by the patient who may be a wage-earner, when he is himself ill, is difficult and often impossible. The essence of such a scheme is that, by small weekly payments when not ill, the contributor shall secure for himself, and possibly for his wife and children, free assistance when he is laid by :—

Important principles arise. The "donor" and the "miserable object" have become one and the same to a certain extent—the poor man becomes a part contributor. The hospital is no longer supported only by the rich for the poor, but becomes to a certain extent a sick club. Certain difficulties at once arise. What will be the position of the Honorary Staff, hitherto unpaid, in this new order of things ? Will the hospital be able to meet its obligations and admit all who ought to be admitted ? Is it jeopardizing its voluntary management ? Should there be an income limit for those who may be allowed to join a contributory scheme ? How will such a scheme affect the general practitioner, and, still more, the consultant ?

### Provincial Contributory Schemes.

Contributory schemes have been in existence for some time in certain provincial towns, and Mr. Langton made inquiries on the spot at Glasgow, Edinburgh, Newcastle, Sunderland, Leeds, Sheffield, Derby, Leicester, Norwich and Oxford. The Glasgow plan is for employés only. There is very little "scheme" in it. It is a "Hospital Saturday Fund" arrangement. The hospitals have no voice in the scheme ; they "honour" the "letters" and take the money, which is systematically collected at the works. Simple as the arrangement is, however, it has very materially increased the income of the four participating hospitals. Nor is there much to learn from Edinburgh. This is not a contributory scheme at all, but a thoroughly well-organised workmen's appeal, without benefit, run by workmen voluntary workers. Its whole spirit is opposed to the Glasgow Superintendent's statement that "the workman expects something for something." The rapid growth in the contributory scheme at Newcastle-on-Tyne is very marked. Practically every workman belongs to it, and is expected to belong to it before asking for the hospital's help. The income from subscriptions and donations at Newcastle Infirmary in 1919 was £13,000, and in the same year from the contributory scheme it was £28,000, which by 1921 had risen to £44,000. Hospital "Appeals," except through the men's representatives, are banned. At Sheffield and Derby employers give one-third of the men's subscriptions annually. At Sunderland employés give £1 per £1,000 of wages paid (with £250 limit). At Leeds, employers give 2s. per worker per annum. A penny a week per worker has been proposed from the employer (Midland Railway's scheme), and the report "rather favours" the Leeds scheme. The Hospital provides the employer with forms, and he fills these up promising subscription at the rate of 2s. per annum for each employee. He secures exemption from Income Tax on these forms.

### A Scheme for the London Hospital.

The report goes on to outline a contributory scheme for the London Hospital. Any suggestion of a "contract of service" must be avoided :—

The London, being a "Charge" Hospital, has something to offer as an inducement in its place, and we think that freedom from charges and from inquiry at the hospital should provide that lure which it is generally agreed is essential to a contributory fund. To strengthen the present position and to accentuate the advantages of a fund, we are of opinion that, while the current scale of "charges" to out-patients might stand, the present "charge" to adult in-patients—21s. a week—should be raised to 35s. a week (as at Sheffield). Children over 7 to remain at 10s. 6d. per week, or to be raised in proportion. Although an income limit for contributors is non-existent in the Provincial centres visited, it is unavoidable at the London, and with a view to the future we suggest the adoption of the King's Fund scale as follows : Individuals, £4 per week, or £208 per annum ; married couples, £5 per week, or £260 per annum ; married with children, £6 per week, or £312 per annum. While we realise that there is much to commend a flat rate of 3d. a week as suggested by the King's Fund, our preference is for a graded rate of 1d. up to £1 of wages, and 1d. for each subsequent completed pound (Sheffield). Non-contributors would naturally be "charged" —as out-patients according to the scale—as in-patients on the 35s. per week charge, in accordance with the income limit—but there should be discretion to regard the "charge" as a datum line and to assess above or below according to circumstances, in the former case even up to full cost of maintenance. Destitute persons and old-age pensioners would be passed free, evidence to be produced when desired. A contributor, his wife, and non-wage-earning children up to the age of 16, provided he be not wilfully in arrears, would be held eligible for free treatment of any disease, &c., for which other arrangements have not been made by Government or other services, and also free from inquiry at the hospital.

Two Concurrent Funds.

While fully realising the widespread value of joint help from employer and employed, and the mutuality of health interests, the authors of the report prefer an actual separation of funds, and would like to see the establishment of an employers' fund made concurrent with the contributory fund :—

Such a fund might well be worked on a capitation basis of 2s. per wagement per annum (Leeds), which, being a definite payment for employees' health, would be a trade expense, and so tax free. We feel that the point may well be raised : What existing income might be affected by the formation of a contributory fund ? Probably some £20,000 to £25,000 would be thus affected, but in the direction of stability, and in any case it would be but a small proportion of what we anticipate a contributory fund should reach. The payment of medical staff is a point not to be overlooked, in view of the recent activity by the British Medical Association. With the single exception of Leicester, we found no provincial hospital with a contributory scheme where a request for payment had not been put forward, and at Leicester, while a percentage was granted on rate, State and approved society payments, it was refused on the contributory scheme funds. It should be noted that the scale of contribution we have suggested does not cover payment of the medical staff, and if fixed payment or a fund percentage were agreed to, the rate would have to be raised to meet a demand which has not been allowed for, and which would be a novelty in hospital history.

### MASS CONTRIBUTIONS IN LONDON.

In our December issue Mr. E. W. Morris set out some of the causes which have brought the Hospital Saving Association into existence and gave an outline of its organisation and proposals. The object of the following notes is to indicate the present position of the Association and to summarise the administrative arrangements which it is making.

The Co-operating Hospitals.

All the hospitals in the Metropolitan Area have been invited to co-operate by formally agreeing for the period of the current year to exempt contributors and dependent children, on production of the certificate of the Association that the contributor conforms to its regulations, from Almoner's inquiries as to means and from request for payment at the hospital. In return co-operating hospitals will receive their proportion of the funds available for distribution, the distribution being based on services rendered to contributors and their dependents. Hospitals have further been invited to nominate " Hospital Members " of the Association on the scale of, roughly, one member to each 50 beds. These " Hospital Members " will be entitled to elect one-third of the Executive Council, the other two-thirds being elected respectively by members representative of contributors and members known as " General Members," representative of the Central Agencies and of the general community. The invitation of the Association has so far been accepted by Guy's, King's College, London, Royal Free, St. George's, St. Mary's, University College, Westminster, Hampstead General, Seamen's (Dreadnought), London Homœopathic, London Temperance and a large number of smaller and special hospitals.

Local Patriotism.

One of the aims of the Association is to foster local patriotism for local hospitals, and it desires to give the fullest play to the individuality of hospitals. With these ends in view, and in order to enable the proposals of the Association to be brought as rapidly as possible before wage earners in the Metropolitan Area, Key Hospitals have been invited to take the lead in initiating branches of the Association. The objects of branches will be to conduct the local business of the Association, to gain and retain local interest in the scheme and in local hospitals, and provide a meeting ground for representatives of local groups of contributors, local hospitals and local employers and residents. As soon as the Key Hospital has intimated that it is ready to move the Associated Hospitals in the area are being informed and invited to co-operate with it. St. George's and the Westminster Hospitals have taken preliminary steps to initiate branches. The London Hospital and others are getting ready to initiate active local campaigns. King's College Hospital has initiated a branch at a well-attended meeting at the Camberwell Town Hall. A resolution was unanimously carried approving the objects of the Association, and a preliminary Committee, consisting of the Mayor and representative employers and employees, was formed to organise a " King's College Hospital " Branch. Arrangements have been made with the National Provident Association under which a group of the Hospital Saving Association will be formed from its members, who will be offered such privileges as are available to contributors to the Hospital Saving Association. The Hospital Saturday Fund has agreed in principle to co-operation and the Chairman of their Executive Committee, Mr. J. F. Stirling, has joined the Provisional Executive Council of this Association. The help of the Fund has been sought and obtained in calculating the proportion of the weekly contribution which is required to be set aside to enable the Association to offer contributors assistance in obtaining surgical instruments, convalescent treatment and other adjuncts to hospital treatment on lines which the Fund has done so much to popularise.

Doing the Spade Work.

Coincidentally with these steps in preparatory organisation a great deal of preliminary spade work has been done in explaining the proposals of the Association to representative men drawn from many trades. This process will be continued and, as an example, at the end of the month Mr. Lesser, President of the National Federation of Employés' Approved Societies and a member of the Executive Council of the Hospital Saving Association, has arranged for Lord Hambleden and Sir Alan Anderson to address their annual meeting. Thus, by the time the Association begins an active campaign of propaganda and recruiting their proposals will have already been widely disseminated among wage-earners. Messrs. W. H. Smith & Son have the distinction of forming the first group, 1,500 strong, the men contributing 2d. weekly and the employer 1d. Contemporaneously the Boards of many of the larger companies are being personally approached.

